# Operating Chairs

**Published:** 1873-06

**Authors:** H. Scott


### OPERATING CHAIRS.

BY H. SCOTT.

EDITOR REGISTER.—I wish to say something about operating chairs, to dentists and chair makers through the REGISTER. I will be brief, but I hope others will concur with me in the suggestions I have to make. I refer to no particular chair ; but I have to say once for all, that I have never yet seen a chair that meets all the requirements, either of the operator or the patient ; and this seems the more strange to me, because the imperfections I have to complain of would be so easily corrected to make chairs exactly what we want.

The back, from the head rest down to the seat at its lowest depression, ought to be as concave, or more so, than most of them are convex. No patient ever did, or ever will sit easily on a chair with a convex back. I suppose that operators generally have uniformly experienced the very annoyances I have myself viz, the patient is in position, and manipulations commenced, and very soon it is found necessary to request the patient in a polite way to sit back in the chair. The reason for this request is, that the sitter has slid forward instinctively to relieve himself or herself from an uneasiness occasioned by the prominence of the upholstering pressing upon the spine, while the neck has to be strained in reaching the rest. And this continues to bear all the same, whether the position of the chair be upright or thrown back. But almost everybody will say " why, every person has a hollow in the small of the back, and the chair ought to be made to fit it." Exactly the reverse of this is true in fact, as any one will easily satisfy himself by sitting down in an old fashioned rocking chair, the back of which is considerably concave ; or by sitting upright in any dentists chair and trying to get the head back squarely on the rest. I have always been annoyed by having to request my patients to sit back in the chair ; or by bending myself down to meet their awkward position. This has been my experience. I have recently employed a workman to alter my chair, (Archers,) but we were unable to bring it to what it ought to be.

The head rest should be one-half longer than usual, and only slightly concave, but never rimmed round, as Archer's are. The ridge like rim made by raising the upholstering round the borders, is a monstrons mistake, as patients heads are half the time, in spite of all the care that can be taken, resting on either the lower or the upper ridge. I could say more that I think would be right to say, but will not at present.

I have submitted these remarks respectfully, and in good faith, as the culmination of careful attention to the matter, and I cannot believe that others will differ much with me.

